# Immune Tolerance as the Physiologic Counterpart of Chronic Inflammation

**DOI:** 10.3389/fimmu.2020.02061

**Published:** 2020-09-30

**Authors:** Vladimir Rogovskii

**Affiliations:** ^1^Department of Molecular Pharmacology and Radiobiology, Pirogov Russian National Research Medical University, Moscow, Russia; ^2^Department of Neuroimmunology, Federal Center of Brain Research and Neurotechnologies, Moscow, Russia

**Keywords:** immune tolerance, chronic inflammation, immune privilege, oral tolerance, cytokines, tumor immunity, inflammation in aging

## Introduction

Chronic inflammation is linked to various diseases, including cancer, diabetes mellitus, obesity, and hypertension. The critical question—how is chronic inflammation linked to acute inflammation and are there any physiological counterparts to chronic inflammation (1, 2). Answers to these questions are of paramount importance as they determine the treatment strategies of diseases associated with chronic inflammation.

In a simplistic approach, it is possible to divide acute inflammation into the two phases: the onset and the resolution (termination of inflammation and return to homeostasis) (3, 4).

Chronic inflammation for various reasons lacks a complete resolution phase—it never ends. There are different reasons for it such as prolonged contact with infection or irritants and the presence of cells that continuously secrete inflammatory mediators. During chronic inflammation, anti-inflammatory cytokines are released continuously—along with pro-inflammatory cytokines (2). So, when the inflammatory stimulus becomes permanent, immunosuppression begins. This property can be used to achieve immune tolerance. For this aim, every immune-privileged site might contain the source of inflammatory factors (5). In this case, slightly elevated levels of inflammatory factors are linked to immune tolerance, while significantly elevated levels are linked to inflammatory exacerbations.

## Examples of Immune Regulatory Roles of Pro-Inflammatory Factors

**Figure 1:**
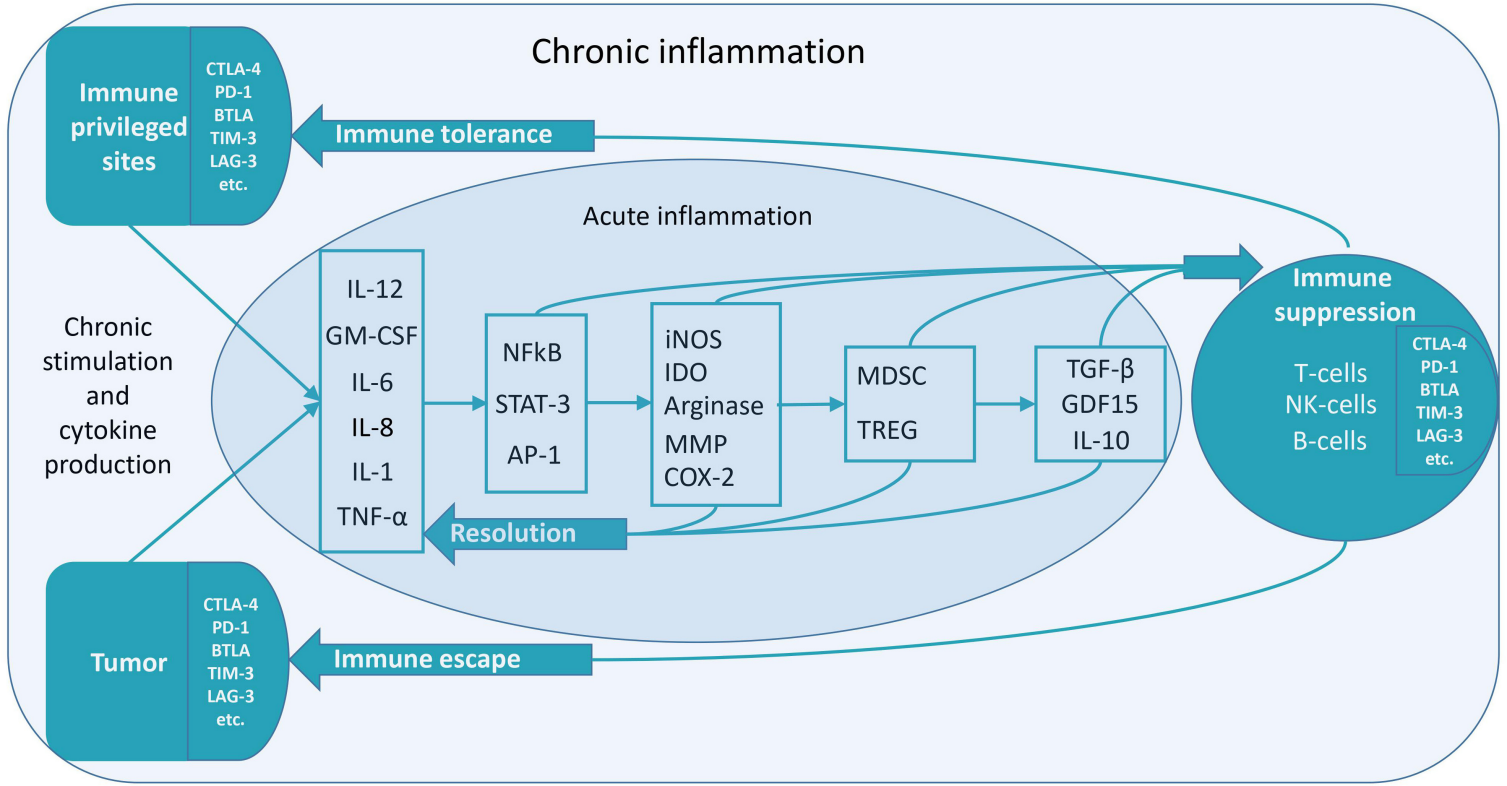
Low-grade constitutive inflammation might be the mechanism of immune tolerance. Various mechanisms of the transition to immune suppression during chronic inflammation exist. The figure depicts some of them. Inflammatory factors induce anti-inflammatory factors, which cause immune suppression (7). In the case of acute inflammation, the resolution of inflammation proceeds without suppressing immunity. It is necessary to stop an acute inflammation in time to prevent its transition to the chronic form. In the treatment of diseases linked to chronic inflammation, there may be two methods to utilize—anti-inflammatory and pro-inflammatory therapy. Both of these therapies are aimed at overcoming the vicious cycle of chronic inflammation. Pro-inflammatory therapy can cause a subsequent anti-inflammatory response, resulting in the resolution of chronic inflammation. For instance, this can be the case in hyperthermia therapy (8). Chronic inflammation, which can occur for various reasons (chronic contact with infection or irritants, chronic stress, and the presence of cells that continuously secrete inflammatory mediators), lacks a complete resolution phase—it never ends. Anti-inflammatory cytokines are released continuously—along with pro-inflammatory cytokines. So, when the inflammatory stimulus becomes permanent, immunosuppression begins. This property can be used to achieve immune tolerance. Chronic low-grade inflammation might be localized—either in immune-privileged organs or tumors. It can lead to immunological tolerance (unresponsiveness to antigens) in the areas of chronic inflammation.

According to recent studies, there is a long-lasting immune post-resolution phase even after acute inflammation, which might be essential for immune tolerance (6). It can be supposed that chronic inflammation significantly enhances this stage. Immune-privileged organs can acquire chronic inflammatory status to maintain immune tolerance. In pathology, e.g., cancer, chronic inflammatory status is also utilized to maintain immune tolerance (Figure 1) (7).

There are various examples of the transition to immune suppression during chronic inflammation.

One such example is the capability of essential inducers of inflammation (specifically, prostaglandins) to function as pro-resolvers—they promote factors necessary for anti-inflammatory and immunosuppressive responses (e.g., specialized pro-resolving mediators) (4). Inflammation also induces growth and differentiation factor 15 (GDF15), which regulate tolerance to inflammatory damage (9).

Another example is that one of the primary inflammatory mediators—IL-6 is needed for regenerative and protective processes in the body. For instance, in mice, IL-6 was essential for liver regeneration, gut barrier repair, and the suppression of inflammation in the kidney and pancreas (2).

The transition of inflammation to immune suppression can also occur at the cellular level. As an illustration, macrophages shut down the generation of pro-inflammatory mediators and activate a transcriptional program resulting in the release of anti-inflammatory cytokines [e.g., IL-10 and transforming growth factor β (TGFβ)] (4).

Other recent studies have shown that long-term activated dendritic cells (DCs) significantly changed their profile toward a non-functional, tumor-promoting, and anti-inflammatory phenotype (10). Such DCs promote the generation of T cells with a regulatory phenotype. One of the mechanisms of DCs turning to tolerogenic cells is the action of immunoregulatory enzymes involved in amino acid metabolism (indoleamine-2,3-dioxygenase 1—IDO1, arginase, and inducible nitric oxide synthase—iNOS). They are induced by chronic inflammation, particularly by repeated stimulation of TLR (e.g., exposure to endotoxin) and are involved in the autoimmunity limitation and maintenance of immune tolerance (11, 12). These enzymes catabolize amino acids causing their deprivation in the microenvironment (arginase and iNOS catalyze the degradation of L-arginine and IDO1 catalyzes the degradation of L-tryptophan) and produce immune regulatory compounds. For instance, IDO1 produces 3-hydroxyantranilic and L-kynurenine, which serve as an activating ligand for the aryl hydrocarbon receptor (AhR) favoring the expression of protective TGFβ, regulatory T cells (Treg cells) differentiation and inducing IDO1 expression in DCs (11, 13, 14).

These mechanisms are involved in endotoxin tolerance—attenuated production of pro-inflammatory cytokines such as tumor necrosis factor-α (TNF-α), IL-6, and IFN-γ, and increased production of anti-inflammatory cytokines such as IL-10 and TGFβ in response to repeated exposure to LPS (lipopolysaccharide) or a gram-negative infection (12, 15).

Above-mentioned enzymes are interconnected as arginase enzymatic activity might be mandatory for the subsequent IDO1 upregulation. Arginine is actively metabolized by arginase to produce urea and l-ornithine. Polyamine spermidine is generated downstream of the decarboxylation of l-ornithine. Spermidine can promote IDO1 phosphorylation and signaling events in DCs, possibly via direct activation of the Src kinase, which has IDO1-phosphorylating activity (16).

## Low-Grade Inflammation in Pregnancy

There is much evidence that low-grade inflammation is significant for maintaining immune tolerance in immune-privileged sites. A successful pregnancy requires fine-tuning the level of inflammation. Either the increase or the decrease in the level of inflammatory mediators leads to negative consequences (7). For instance, it was shown that both a decrease or increase in the IL-6 concentration enhances the risk of infertility and miscarriage (17).

According to recent studies, it might be proposed that chronic moderate antigen stimulation might be necessary for successful immune tolerance in pregnancy as the repeated LPS exposure leads to placental endotoxin tolerance (18).

Pregnancy, especially implantation, evokes an inflammatory reaction, which includes the upregulation of inflammatory cytokines [e.g., IL-6, IL-1, leukemia inhibitory factor (LIF)]—they are critical mediators of a healthy pregnancy. Besides, various leukocytes are found in the decidua, including maternal natural killer (NK) cells, DCs, macrophages, and lymphocytes (19).

The specific subset of NK cells, which constitutes about 50–90% of total lymphoid cells in the uterus, plays a vital role at the fetal-maternal interface during the first trimester of the pregnancy (20).

HLA-G molecules are considered to be crucial for the immunological tolerance of the fetus by the mother. At the same time, they may be involved in the active secretion of pro-inflammatory cytokines (21, 22). Another example—the granulocyte-macrophage colony-stimulating factor (GM-CSF), which is involved in myeloid cell development and inflammation (23). Recently, Chu et al. (24) have shown that GM-CSF has a beneficial effect on the development of human embryos in assisted reproductive technology.

Kieffer et al. (25) have shown that healthy pregnancies have a higher activation of CD4+ memory T cells compared to preeclampsia.

Due to the possible tolerogenic role of low-intensity inflammation, anti-inflammatory therapy can reverse the level of immune tolerance factors. As an example of this in mice, prednisolone, known as an immune suppressor, disrupted the expected expansion of CD4+ T cells in early pregnancy, inhibiting the generation of both suppressive Treg cells and effector T cells (26).

Other anti-inflammatory substances, nonsteroidal anti-inflammatory drugs (NSAIDs), are known for their serious fetal side effects. NSAIDs inhibit the production of prostaglandins, which are essential for successful embryonic implantation (27). Prostaglandins can cause low-grade inflammation in pregnancy (e.g., in the decidua), which can help to induce the immune tolerance necessary for normal fetal development.

## Low-Grade Inflammation in Other Immune-Privileged Sites—The Central Nervous System and Oral Tolerance

According to the concept of the necessity of low-grade physiological inflammation for maintaining immune tolerance, every immune-privileged organ should contain the source of inflammatory factors.

In the central nervous system, glial cells, along with the neurons, can act as the source of inflammatory factors in a steady-state (28). A certain level of IL-1 is detected in the healthy brain where it exerts a neuro-modulatory role. Microglia is the primary source of IL-1 production in the brain without infiltrated leukocytes. When the IL-1 level is increased over a certain threshold level, it becomes associated with various neuroinflammatory conditions (29).

Basal IL-6 and IFN-γ levels in the healthy brain are also linked to the maintenance of brain homeostasis (30). IFN-γ is involved in IDO induction, the immune regulatory properties mentioned above (11, 31). IFN-γ is also the upstream regulator of iNOS expression. According to various studies, iNOS may have a regulatory function during neuroinflammation and autoimmunity (in addition to its pro-inflammatory role) (32). Besides its pro-inflammatory role, IL-6 is the inductor of suppressive Treg cells. According to the recent work of Hagenstein et al., engagement of the IL-6 receptor leads to the generation of a unique Treg subtype with enhanced suppressive capacity. These cells express transcription factor RORγt—similar to pro-inflammatory Th17 cells (33–35).

Interestingly, cytokines that are associated with the induction of neuroinflammation in some cases can exert a protective effect. For instance, the administration of IL-12 (which amplifies Th1 polarization) during the early phases of experimental autoimmune encephalomyelitis (an animal model of multiple sclerosis) suppressed the disease through the induction of IFN-γ (36, 37). IL-8 levels (cerebrospinal fluid, as well as serum) were found to be significantly lower in Alzheimer's disease patients (38). Reduced IL-8 is also linked to HIV-associated neurocognitive disorder (39).

Brain NK cells control the inflammation by killing pro-inflammatory microglial cells, which are activated within minutes of ischemia onset (40).

NK cells are a major lymphocyte subpopulation within the rat testis (41). Sertoli cells, spermatocytes, and round spermatids are known to produce pro-inflammatory IL-1α (42).

According to modern approaches, the traditional labels of pro- and anti-inflammatory are too simplistic to describe cytokine actions. Genetic ablation of IL-17 (overexpressed in the inflamed intestine as a contributor to intestinal damage) declined gut epithelial cell proliferation and exacerbated dextran sulfate sodium (DSS)-induced murine colitis (43). Overall, data suggest both a pro-inflammatory and a protective function of the IL-17 immune response (44).

Generally, in recent years we have been faced with a revision of the typical inflammatory role of inflammatory factors (33, 45). For instance, the physiological role of GM-CSF is to amplify adaptive immunity and inflammatory responses by recruiting DCs (45). Nevertheless, these DCs might be either inflammatory or tolerogenic. According to recent studies, the microbiota-dependent generation of the GM-CSF is of paramount importance for DC-mediated oral tolerance, and this factor in itself has previously been linked to Crohn's disease development (46, 47).

Diet and gut flora metabolites promote the ability of DCs to control T cell responses in the gut and other sites (e.g., lungs) (48, 49). Thus, immune stimulation by microbiota antigens (e.g., LPS) can mediate low-intensity physiological inflammation in the intestine, which is necessary for intestinal mucosal tolerance (50).

## Elevated Inflammation in Aging And Immune Tolerance

In contrast to younger individuals, older individuals have elevated levels of inflammatory cytokines, especially IL-6 and TNF-α (51, 52). Fat tissue might be the main source of inflammatory cytokines in aged people. Adipocytes can produce pro-inflammatory and chemotactic compounds, such as IL-6, IL-1β, and TNF (52, 53). IL-6, TNF-α, and their receptors are also upregulated in other aged tissues and cells (54).

The increased basal level of inflammatory cytokines can mediate increased immune tolerance. That might be the reason why older persons have higher autoimmunity but a lower prevalence of autoimmune diseases (55). The price of increased immune tolerance is increased susceptibility to tumors (56).

Young individuals have decreased basal levels of various inflammatory cytokines—this may explain a reduced immunological tolerance, and, consequently, higher susceptibility to autoimmune diseases, which is observed in young people (57). Progressive increases in the percentage of total lymphocytes and absolute numbers of T and B cells in infants compared to adults might be evidence of the reduced immunological tolerance in young individuals (51).

## Cytokines Level and Immune Tolerance

As we have previously shown, constitutive slightly elevated cytokine levels may be linked to immune tolerance. These are the same cytokines whose levels rise significantly during inflammatory and autoimmune disorders (e.g., IL-6, IL-1, and IFN-γ). The main difference between these two conditions is the cytokine concentration—slightly elevated in immune tolerance and significantly elevated during exacerbations.

According to the latest research, a very low cytokine level is sufficient to develop a specific effect. For example, binding of as few as 4 IL-6 molecules per cell seemed to result in statistically significant bioactivity, whereas binding of 16 IL-6 molecules triggered extensive cellular responses (58).

The cytokine profile of physiological fluids related to immune-privileged organs (e.g., eye, placenta, central nervous system, and testes) is of significant interest. It may reflect the constitutively increased content of cytokines in the tissues of these organs.

For instance, the GM-CSF level in plasma of healthy adults is about 2.5 pg/ml while the GM-CSF level in tears (this fluid is related to ocular immune privilege) of healthy subjects is about 30 pg/ml (59, 60). The average physiological concentrations of IL-6 in human serum are 1–5 pg/ml. In comparison, the IL-6 level in tears of healthy subjects is more than 100 pg/ml (59, 61). The plasma concentration of IL-1β in healthy controls is about 2.5 pg/ml, while the IL-1β level in tears of healthy subjects is about 100 pg/ml (59, 62). At the same time, in infectious inflammation, the levels of these cytokines in tears sometimes increase by more than 10 times (63).

In other physiological fluids related to immune-privileged sites, we can also observe a slightly increased level of specific pro-inflammatory cytokines: for example, in amniotic fluid, CSF (especially IL-8), and semen (64–68). In the case of inflammatory diseases in these organs, the level of these cytokines rises significantly. Thus, we can distinguish three types of inflammation (the level of inflammatory factors)—low (normal or absent), medium (constitutive low-grade inflammation—associated with immune tolerance), and high—associated with an intense immune response.

Perhaps, IL-1β, IL-6, and GM-CSF might be some of the most critical cytokines for tolerance induction. These cytokines are also involved in the induction of immune-suppressive myeloid-derived suppressor cells (MDSCs) by various cancer cell lines (69).

## Conclusion and Prospects

As already mentioned, there is an increased level of some inflammatory cytokines in immune-privileged sites, compared with blood plasma. Inflammatory cytokines are also critical at the beginning of the resolution phase of inflammation.

Can we talk about physiologic low-grade inflammation, which is linked to immune tolerance, as the physiologic counterpart of chronic inflammation (or para-inflammation)? (1, 70).

There might be various consequences of this hypothesis—for example, in violation of the diversity of microbiota antigen stimulation (specifically, due to the use of antibiotics)—autoimmune lesions may occur (71). Another consequence is the new argument in favor of anti-inflammatory therapy in cancer. Can such therapy reduce the tolerance to tumor antigens? (5) Also, in the treatment of diseases linked to chronic inflammation, there may be two ways to tackle it—anti-inflammatory and pro-inflammatory therapy. Both of these therapies are aimed at overcoming the vicious cycle of chronic inflammation (Figure 1).

Thus, the maintenance of chronic low-grade inflammation might be the universal mechanism of immune tolerance. The basement of such physiologic low-grade inflammation might be in naturally increased pro-inflammatory cytokine secretion. This process can be observed both in disease (e.g., cancer) and in health (immune-privileged sites) (5).

It is well-known that chronic exposure to antigens may cause not only immune tolerance but also cause an excessive immune reaction, including autoimmunity (15, 72–74). Thus, that raises another important question—how does immunity determine which method of interacting with antigens should be used - immune tolerance or elimination? It might be proposed that besides the discrimination of self and non-self, immunity makes another critical decision—dangerous or not dangerous, and applies immune tolerance to antigens deemed as not dangerous. The exact mechanisms of this choice remain to be fully elucidated.

## Author Contributions

The author confirms being the sole contributor of this work and has approved it for publication.

## Conflict of Interest

The author declares that the research was conducted in the absence of any commercial or financial relationships that could be construed as a potential conflict of interest.
